# Supplementary material for: Geographic Location, Population Dynamics, and Fruit Damage of an Invasive Citrus Mealybug: The Case of Delottococcus aberiae De Lotto in Eastern Spain
Source: Insects. 2024 Aug 30;15(9):659. doi: 10.3390/insects15090659 (PMC11432470; doi:10.3390/insects15090659)
Supplement: Supplementary file 1 [file insects-15-00659-s001.zip › insects-3164461-supplementary/Supplementary F2 mod.pdf]

Supplementary Materials 2

For each month, male catches were compared among the study years (2019-2023) by means of a One-way ANOVA (Fisher least significant difference [LSD] test at $P < 0.05$). Trapping data (males per trap and day, MTD) were was log-transformed (ln[captures+1]) to normalize residuals data distribution and homogenizing the variance. The resulting statistics are shown in the following Table S1. These correspond to the information depicted in Figure 3 of the manuscript.

Table S1. Statistics of the Fisher LSD test ($P < 0.05$) after the ANOVA analysis applied to compare the number of male catches (MTD) recorded on the 58 or 78 traps of the monitoring network each month among the study years.

**Month**	**F**	**df**	**P**
January	42.88	164,2	<0.0001
February	22.80	164,2	<0.0001
March	21.94	212,3	<0.0001
April	3.01	208,3	<0.05
May	4.74	271,4	<0.01
June	2.84	274,4	<0.05
July	1.97	279,4	0.09
August	8.57	275,4	<0.0001
September	8.50	255,4	<0.0001
October	0.55	272,4	0.70
November	0.53	275,4	0.71
December	10.56	167,2	<0.0001
