# Supplementary material for: Geographic Location, Population Dynamics, and Fruit Damage of an Invasive Citrus Mealybug: The Case of Delottococcus aberiae De Lotto in Eastern Spain
Source: Insects. 2024 Aug 30;15(9):659. doi: 10.3390/insects15090659 (PMC11432470; doi:10.3390/insects15090659)

Figure S1. *Delottococcus aberiae* male catches recorded at each of the 78 sampling units of the monitoring network from January to Septembre in each study year (2020-2023). Captures were obtained in pheromone-baited monitoring sticky traps (males/trap/day) and were represented according to the following scale:): 0 (white dot), 0-1 (light green dot), 1-3 (dark green dot), 3-6 (yellow dot), 6-10 (light orange dot), 10-20 (orange dot), > 20 (red dot).

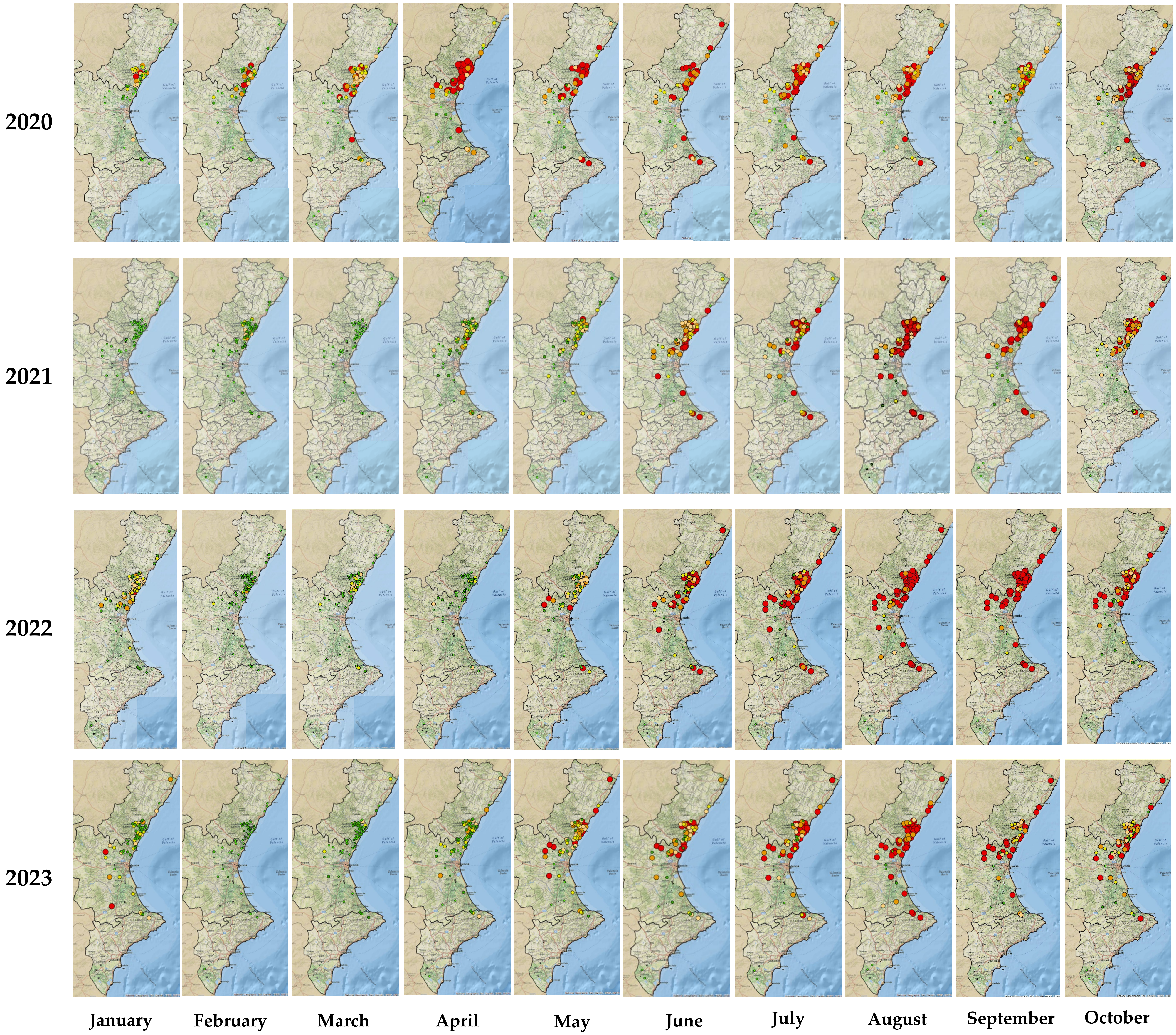

Supplement: Supplementary file 1 [file Supplementary F1 DistribucionMonitoring.pdf › insects-3164461-supplementary/Supplementary F1 DistribucionMonitoring.pdf]
